# Investing in midwifery for sustainable development goals in low- and middle-income countries: a cost-benefit analysis

**DOI:** 10.1186/s12962-023-00507-y

**Published:** 2024-01-04

**Authors:** Chakib Boukhalfa, Brahim Ouakhzan, Hanane Masbah, Leila Acharai, Saad Zbiri

**Affiliations:** 1 National School of Public Health, Rue Lamfadel Cherkaoui, Madinat Al Irfane, Rabat, BP 6329 Morocco; 2 Human Resources Direction, Ministry of Health and Social Protection, Rabat, Morocco; 3 United Nations Population Fund, Rabat, Morocco; 4 International School of Public Health, Mohammed VI University of Sciences and Health, Casablanca, Morocco; 5 Laboratory of Public Health, Health Economics, and Health Management, Mohammed VI Center for Research and Innovation, Rabat, Morocco

**Keywords:** Cost-benefit analysis, Investment, Midwifery, Sustainable development goals, Maternal and child health, Morocco

## Abstract

**Background:**

Maternal and neonatal mortality in low- and middle-income countries is frequently caused by inadequate management of obstetric and neonatal complications and a shortage of skilled health workers. The availability of these workers is essential for effective and high-quality healthcare. To meet the needs of sexual, reproductive, maternal, new-born, child, and adolescent health by 2030, more than one million health workers, including 900 000 midwives, are required globally. Despite this, uncertainty persists regarding the return on investment in the health workforce.

**Methods:**

The objective of this research was to determine the cost-benefit ratio of increasing investment in midwifery in Morocco from 2021 to 2030. A comparative analysis was conducted between scenarios “with” and “without” the additional investment. The costs and benefits were estimated using relevant data from national and international sources.

**Results:**

Following the International Confederation of Midwives’ recommendations, it is advised that Morocco recruit 760 midwives annually to achieve 95% of universal health coverage. This increase in midwifery could result in saving 120 593 lives by 2030, including reducing maternal deaths by 3 201, stillbirths by 48 399, and neonatal deaths by 68 993. The estimated economic benefit of investing in midwives was US$ 10 152 287 749, while the total cost was US$ 638 288 820. Consequently, the cost-benefit ratio was calculated as 15.91, indicating that investing in midwifery would provide 16 times more benefits than costs.

**Conclusion:**

Increasing investment in midwifery appears to be an efficient strategy for achieving comprehensive maternal and child health coverage in low- and middle-income countries.

## Introduction

While most countries worldwide have made progress in reducing maternal and infant mortality rates, many low- and middle-income countries (LMICs) continue to have rates above the recommended thresholds set by the sustainable development goals (SDGs). These goals are expected to be met by all countries by 2030 [1].

Most maternal, foetal, and neonatal deaths occur due to inadequate or delayed management of preventable complications [2]. Providing appropriate and quality care requires an adequate number of healthcare workers who are motivated and have the necessary skills [3, 4]. To achieve the sexual, reproductive, maternal, new-born, child, and adolescent health (SRMNCAH) goals by 2030, over a million new health workers, including 900 000 midwives, are needed [5, 6]. The COVID-19 pandemic has worsened the situation, leading to the relocation of many health workers to fill vital gaps in other health services [6].

As a result, LMICs need to address the shortage of midwifery care to improve maternal and child health. Only a small percentage of women require a doctor’s presence during childbirth, and midwives can provide a range of services beyond delivery, such as pregnancy and postpartum care, family planning, cancer screening, and HIV control [6]. Deploying midwives is critical for strengthening the health workforce and achieving the SDGs related to maternal and child health. Therefore, investing in the midwifery workforce to ensure access to quality care is essential for health system performance.

In Morocco, the maternal and neonatal situation has improved in recent years. Based on the 2018 national population and family health survey, maternal mortality ratio reduced by 35%, from 112 maternal deaths per 100 000 live births in 2010 to 72.6 in 2018, and neonatal mortality rate reduced by 37%, from 21.7 per 1 000 live births in 2010 to 13.6 in 2018. However, despite the improvements, there are still significant differences between geographic and socioeconomic population groups, with higher maternal mortality rates in rural areas compared to urban areas [7].

Economic evaluation remains one of the best strategies in examining the efficiency of health programs, particularly through cost-benefit analysis. Such analysis can be crucial for mobilizing resources and convincing stakeholders that spending on midwifery is an investment rather than just a cost. Economic arguments may resonate well with stakeholders who influence health investment decisions [8].

To date, no economic study exists on the return on investment in midwifery in Morocco. The question that still remains unanswered is: What is the cost-benefit of investing in midwifery in Morocco to achieve the SDGs, particularly goals 3 and 5? The objective of this study was to calculate the cost and benefits of investing in midwifery in Morocco and estimate the resulting cost-benefit ratio. One of the main gaps in research into the return on investment of midwifery in Morocco is the lack of comprehensive and timely data. Another gap is that quantifying benefits accurately can be difficult. To fill these gaps, our study conducted in-depth research and collected quality data specific to midwifery. In Morocco, the current number of midwives is estimated at around 5 700 midwives, based on the State of the World’s Midwifery (SoWMy) report [9]. Midwives are more concentrated in large cities, and urban versus rural areas [10]. To ensure universal health coverage, Morocco should have around 14 000 midwives by 2030 [9].

## Materials and methods

The study followed a multi-phase analysis approach to meet its objectives, which included data collection, cost and benefit estimation, financial impact assessment, and cost-benefit analysis.

### Data collection

The first step was to identify midwifery strategies in Morocco and benchmark with other countries comparable to Morocco that have already invested in midwifery. The triad of interventions recommended by the International Confederation of Midwives (ICMs) was used to estimate the cost of investing in midwifery care. This triad of interventions is based on three interconnected pillars: training, regulation, and associative work [5, 6]. In the second step, the financial impact of investing in midwifery was estimated by considering the reduction in maternal and neonatal mortality and the increase in the gross domestic product (GDP). Finally, a cost-benefit analysis was conducted to compare the outcomes of investment in midwifery with and without such investment. The net present value was also estimated.

Specific data sources were used to estimate the costs and benefits of investing in midwifery in Morocco. For costs, we used the latest budgetary statements from the Ministry of health and training centers to estimate direct costs, including personnel and training expenses. For benefits, we used the latest national population and family health survey [7], and the Lancet series on midwifery care [11], to estimate the potential number of saved women and infants, the economic report on Morocco of the World Bank Group to estimate the GDP rate [12], the confidential survey of maternal deaths in Morocco to estimate the life expectancy of women and infants [13], and the World Health Organization’s (WHO) recommendation to consider the social interest rate to calculate future values [14].

### Data estimation and analysis

The study conducted an annualized ex-ante economic analysis to evaluate the incremental costs and additional benefits of investing in midwifery in Morocco. The incremental cost is the additional cost of investment, and the incremental benefit is the monetary value of reduced maternal and neonatal mortality. The estimates were converted to US dollars using the exchange rate of the US dollar to Moroccan dirham (1 US$ = 8.94 MAD). The cost-benefit analysis measures the financial impact of an investment by comparing its incremental costs with its incremental benefits in monetary units, using the cost-benefit ratio, as follows:

$$ CBR = \Delta B/\Delta C = {B_1} - {B_0}/{C_1} - {C_0}$$

Where CBR is the cost-benefit ratio, B is the benefit, and C is the cost.

This study used a comparative approach to measure the macroeconomic benefits of investing in midwifery. The “with” scenario aimed to highlight the positive impact of the investment on the well-being of communities, while the “without” scenario examined the economic impact of maintaining the status quo without additional investment.

The investment in midwifery in Morocco is projected to save numerous lives and enhance the quality of life for women and new-borns, leading to long-term benefits in socioemotional functions, health-related behaviours, and economic self-sufficiency. The total economic benefits are expected to be substantial. The study estimated the present value of the investment’s benefits, which are mainly categorized as the reduction in maternal deaths and stillbirths/neonatal deaths. Finally, the cost-benefit ratio was calculated based on the anticipated benefits of investing in midwifery.

#### Estimation of costs

Midwifery care involves training, regulation, and associative work. Morocco has made progress in these areas, including the implementation of a competence-based approach and a law regulating midwifery practice. These achievements have a cost, which is considered the initial cost (C_0_). The additional investment (C_1_-C_0_) represents the estimated cost of creating new budgetary positions for the staff needed for SRMNCAH by 2030, including training and capacity building. These costs are indicated for a 10-year period from 2021 to 2030, and are necessary to achieve SDGs 3 and 5.

To ensure accurate cost estimates for investing in midwifery, this study used the latest budgetary statements from the Ministry of health and training centers including schools of midwifery and nursing to estimate direct costs such as personnel and training expenses. Budgetary statements were used to avoid errors that could arise from differences between estimated and actual data.

The study updated the cost of the investment using the WHO’s recommended social interest rate of 3% to calculate future values [14]. We used the recosting equation as follows:

$$ FV = PV \times {\left( {1{\text{ }} + {\text{ }}r} \right)^n}$$

Where FV is the future value, PV is the present value, r is the discount rate, and n is the number of time periods.

The future values of the realized benefits were also updated as follows:

$$ PV = FV \times {\left( {1{\text{ }} + {\text{ }}r} \right)^{ - n}}$$

Where PV is the present value, FV is the future value, r is the discount rate, and n is the number of time periods.

#### Estimation of benefits

Investing in maternal health is crucial for public health as it not only leads to healthy families but also affects children’s education and long-term productivity growth. By investing in midwifery, the short-term benefits include reducing maternal and child mortality rates, while the long-term benefits include an increase in GDP at the macro level [15]. The reduction in maternal and infant deaths was estimated by comparing the expected number of deaths with and without the investment, based on the current health status and mortality rates of the population [7]. The increase in GDP was calculated using the human capital method, based on estimates from the World Bank Group’s Morocco economic report 2021 [12]. Investments in midwifery have a wide range of impacts on women and infants, including biological, financial, social, and psychological effects [11]. According to the SoWMy-2021 report, investing in midwives can improve health outcomes, promote workforce growth, encourage economic empowerment, and have positive macroeconomic consequences [6].

The study aimed to investigate the impact of skilled personnel on reducing maternal and neonatal deaths. The benefit estimates were based on actual survivors and deaths, and excluded secondary benefits while including reduced mortality rates from previous studies [16, 17]. The total benefits were estimated by multiplying the number of survivors with the GDP per capita and the expected life expectancy in Morocco. The number of survivors was estimated by comparing the expected number of deaths with and without the midwifery investment. Using data on the average age of pregnant women, the expected life expectancy in Morocco was estimated for women and infants [13]. We estimated the benefits per person per year on the basis of constant GDP per capita in 2030 [12]. We then calculated future benefits using a social discount rate of 3% [14].

The scientific literature recommends three scenarios for coverage of midwifery interventions to reduce maternal, stillbirth, and neonatal mortality. The scenarios suggest an increase in coverage by 10% every three to five years, 25% every three to five years, or 95%. A Lancet series on midwifery care examined the impact of these scenarios on 78 countries, which were divided into three country groups based on their human development index. Morocco is part of group C [11], which includes countries with a moderate to high human development index (Table 1).

**Table 1:** Reduction in deaths according to different scenarios for Morocco

		Maternal deaths^a^	Stillbirth^b^	Neonatal deaths^b^
**Current situation**	No variation	72	14	14
**Scenario 1**	10% increase every 3 to 5 years	↘ 62.7%	↘ 50.1%	↘ 52.5%
**Scenario 2**	25% increase every 3 to 5 years	↘ 68%	↘ 51%	↘ 63.6%
**Scenario 3**	95% universal coverage	↘ 69.8%	↘ 52.9%	↘ 77.4%

^a^ per 100 000 live births; ^b^ per 1 000 live births

Source: Lancet 2014 [11]

To improve the measurement of the actual availability of health personnel, we used full-time equivalents (FTEs) to assess the number of qualified staff and the time they spend providing SRMNCAH services.

#### Estimation of cost-benefit ratio and net present value

Based on estimates of the additional investment and the additional benefits of investing in midwifery, the cost-benefit ratio was estimated. When the cost-benefit ratio is greater than 1, it means that the expected benefits are greater than the expected costs. This is generally considered a positive indicator, as it suggests that the investment should generate a positive return on investment. The net present value was also calculated, which involves expressing the consequences of the investment in monetary terms relative to the cost of the investment. Indeed, the difference between the present value of all cash inflows and all cash outflows of the study was calculated. The profitability of the investment was determined by comparing the present value of expected revenues to the amount of capital invested. The net present value equation used is as follows:

$$ NPV = T\sum n = 1{C_t}{\left( {1 + r} \right)^n} - {C_0}$$

Where NPV is the net present value, C_t_ is the net cash inflow in period t, C_o_ is the total initial investment cost, r is the discount rate, and n is the number of time periods.

## Results

### Cost estimates

The study discusses the staffing levels and projections for midwifery care in Morocco based on the SoWMy-2021 report. The report recommends that Morocco should have about 14 000 midwives by 2030, while the current number of midwives is around 5 700 [9]. The study suggests that the universal coverage scenario, which requires the recruitment of an average of 760 midwives per year, is the most attractive for Morocco to accelerate the reduction of maternal, stillbirth, and neonatal mortality by 2030. The first two scenarios require a maximum of 420 midwives per year by 2030 [9].

### Investment in the recruitment of new health professionals

#### Additional recruitment of midwives

Universal coverage of 95% of interventions recommends the recruitment of 13 300 midwives, but currently, there are only 5 704 midwives, resulting in a shortage of almost 7 600 midwives. The cost of recruiting 7 600 additional FTE midwives between 2021 and 2030 is estimated to be US$ 26 239 651.34, with an average gross monthly salary of US$ 898.55 for a newly recruited midwife (Table 2). However, midwives cannot provide all SRMNCAH services alone and work in a multidisciplinary team with other health professionals such as obstetrician-gynaecologists, nurses, and community health workers, who should also be considered.

**Table 2:** Estimated costs of additional investment in midwifery

Additional investment	Cost (US$)
Recruitment of midwives	26 239 651
Recruitment of obstetrician-gynaecologists	446 300
Recruitment of nurses	1 187 821
Recruitment of community health workers	5 989 124
Initial training	87 951 654
Continuous training	8 567 864
Simulation centers	867 611
Staff practice until retirement	507 038 794
**Total**	**638 288 820**

#### Additional recruitment of obstetrician-gynaecologists

The recommended number of obstetrician-gynaecologists is 100, and the SoWMy-Morocco 2021 indicates an FTE rate for this category of 50%. Therefore, there is a need for 200 FTEs by 2030. The average gross monthly salary of a newly recruited obstetrician for the period 2021–2030 is US$ 1 907.63. The future value of the investment required to recruit the additional 200 FTEs is estimated to be US$ 446 300 (Table 2).

#### Additional recruitment of nurses

SoWMy-Morocco 2021 indicates an FTE rate for this category of 44%. The recommended number of nurses by 2030 is 1 136 FTEs. The future value of the investment in the additional recruitment of 1 136 nurses for the period 2021–2030 is US$ 1 187 820.92, with an average gross monthly salary of US$ 898.55 for a newly recruited nurse (Table 2).

#### Recruitment of community health workers

Morocco currently has no community health workers in SRMNCAH, but the SoWMy-Morocco 2021 recommends hiring 4 050 community health workers for this category by 2030. The average gross monthly salary wage for a community health worker is US$ 318.79 for the period 2021–2030. The future value of the investment in recruiting these additional workers is estimated to be US$ 5 989 124 (Table 2).

### Investment in initial training of midwives

The cost of midwifery education in Morocco involves several expenses, such as the cost of teaching staff, administrative staff, operating expenses, and supervision of trainees.

The cost of teaching staff for a student midwife is US$ 1 086.80 for the first year, US$ 1 311.85 for the second year, and US$ 1 423.04 for the third year. Administrative staff cost is US$ 15 per student, and operating expenses cost is US$ 482.10 per student per year. Practical training is conducted at primary health care centers and hospital departments, with one tutor for every four student midwives. The cost of midwifery education for a student midwife is US$ 2 523.49 for the first year, US$ 3 956.60 for the second year, and US$ 4 604.70 for the third year, totalling US$ 11 084.79 for three years of midwifery education.

The depreciation expenses for the schools of midwifery and nursing have been fully depreciated since the fixed assets are aged and the equivalent annual cost of the initial capital expenditure is zero. Therefore, the total cost of the initial midwifery education for 7 000 midwives is US$ 87 951 653.58, with 600 midwives already graduated and inactive (Table 2).

### Investment in continuous training of midwives

The Ministry of health in Morocco recommends an integration course for all newly recruited midwives, which includes 15 days of paid theoretical training and two and a half months of unpaid practical integration training. This course is aimed at equipping the midwives with the necessary skills before they are assigned to their final positions. This recommendation applies to the new midwives to be recruited. The cost of the integration course includes the average cost of accommodation, food, and supplies. The average cost of accommodation is US$ 100.67 per night, the average cost of food is US$ 22.37 per day, and the cost of supplies is US$ 2.80 per person. Therefore, the total investment cost for the integration course for the midwives is US$ 8 567 863.87 (Table 2).

### Investment in simulation centers

To cover the whole national territory, Morocco needs to invest in acquiring specific equipment for the other eight regions, as only four schools of midwifery and nursing have simulation centers. The cost of purchasing equipment for a simulation center for gynaecology-obstetrics ranges between US$ 89 485.45 and US$ 111 856.82, and the future value of this investment is estimated at US$ 867 611.18 (Table 2).

### Investment until retirement

As the retirement age in Morocco is 63 years, recruited midwives will potentially work for an estimated 40 years until retirement, and the future value cost of the investment for the 30 years after 2030 and before retirement is US$ 425 134 947.32 for midwives, US$ 16 639 887.58 for obstetrician-gynaecologists, and US$ 65 263 959.50 for nurses. The total future value cost of the investment after 2030 and before retirement for the 7 000 midwives, 200 obstetrician-gynaecologists, and 1 136 nurses to be recruited for SRMNCAH is US$ 507 038 794.41. The total additional investment includes the costs of new budgetary positions, initial training, and capacity building through continuous education, which is US$ 638 288 820 as per Table 2.

### Benefit estimates

The investment in midwifery covers prenatal, perpartum, and postpartum care, which is expected to reduce maternal and infant mortality rates in the short term. Additionally, the national economy is projected to grow in the medium to long term with an increase in population. The benefits of the investment include a reduction in maternal and infant deaths, which were estimated by comparing the expected number of deaths with and without the investment program. To estimate the expected number of deaths without the investment, the Lives Saved Tool (LiST) developed by the Child Health Epidemiology Reference Group (CHERG) was used based on the current health status and mortality rates of the population [11].

Morocco has maternal, stillbirth, and neonatal mortality rates of 72 per 100 000 live births, 14 per 1 000 live births, and 14 per 1 000 live births, respectively [7]. With the recruitment of the recommended number of midwives by the ICMs and with 95% universal coverage, there will be a significant reduction in maternal, stillbirth, and neonatal mortality rates. The estimated reduction rates are 69.8%, 52.9%, and 77.4% respectively (Table 1). The number of survivors is 3 201 in terms of reduced maternal deaths, 48 399 in terms of reduced stillbirths, and 68 993 in terms of reduced neonatal deaths. By achieving 95% universal coverage, 120 593 lives could be saved, representing 9 360 000 life years. The study used an average age of 31.53 years for pregnant women, obtained from data collected in six priority regions in Morocco [13].

Using data on age and life expectancy in Morocco, we estimated that the benefits of the investment in midwifery would last for 46 years for women and 78 years for infants. We calculated the benefits per person per year based on the constant GDP per capita (US$ 4 795) in 2030, considering the economic impact of the COVID-19 pandemic in 2020 [12]. To estimate future benefits, we applied a social discount rate of 3% [14].

Table 3 presents the estimated post-implementation benefits of investing US$ 638 288 820 in midwifery. The total benefit is estimated to be US$ 17 686 912 454, with US$ 384 860 117 gained from saved women and US$ 17 302 052 337 gained from saved infants. These estimations were calculated based on the number of saved women and infants, the GDP rate, life expectancy at birth, and the discounted accumulated benefits.

**Table 3:** Estimated benefits of additional investment in midwifery

Benefits	Number of survivors	Benefit period	Monetary value (US$)
Reduction of maternal mortality	3 201	46	384 860 117
Reduction of stillbirths	48 399	78	7 133 417 076
Reduction of neonatal mortality	68 993	78	10 168 635 261
**Total**			17 686 912 454

Investing in universal coverage of maternal and infant health interventions can avoid a significant percentage of maternal deaths, stillbirths, and neonatal deaths, resulting in a benefit of US$ 17 686 912 454. Midwives are effective in reducing these deaths and can prevent about 70% of them [11]. However, only 82% of the needs are met by midwives according to SoWMy-2021 report [9]. Investing in midwifery alone could thus result in a benefit of US$ 10 152 287 749 (Table 4).

**Table 4:** Estimated cost-benefit ratio of additional investment in midwifery

Additional investments	Cost (US$)
Recruitment of midwives	26 239 651
Recruitment of obstetrician-gynaecologists	446 300
Recruitment of nurses	1 187 821
Recruitment of community health workers	5 989 124
Initial training	87 951 654
Continuous training	8 567 864
Simulation centers	867 611
Staff practice until retirement	507 038 794
**Total 1**	**638 288 820**
**Investment benefits**	**Monetary value (US$)**
Reduction in maternal and new-born mortality (A)	17 686 912 454
Interventions performed by the midwife (B = 70% of A)	12 380 838 718
Potential to meet needs (82% of B)	10 152 287 749
**Total 2**	**10 152 287 749**
**Cost-benefit ratio**	
Total 2 over Total 1	**15.91**

### Cost-benefit estimates

The study calculated the cost-benefit ratio of the additional investment in midwifery in Morocco, which is a useful indicator in studying project investments. The economic benefit that could be obtained from the investment is US$ 10 152 287 749, and the total cost is US$ 638 288 820. The cost-benefit ratio is 15.91 ≈ 16, indicating that the investment in midwifery is 16 times more likely to provide benefits than costs (Table 4). The net present value is over US$ 9.5 billion, which confirms that the investment is profitable. Indeed, when the cost-benefit ratio is greater than 1, this indicates that the benefits outweigh the costs. A higher ratio suggests a more favourable investment. In our study, a ratio of 15.91 is higher. This means that the investment will contribute to the production of net economic value. This can be beneficial for the whole of society.

This result has several implications for health policy and planning. To help achieve development goals related to the reduction of maternal and infant mortality, the main recommendation is to increase the recruitment of midwives and other health professionals involved in the SRMNCAH. In addition, it would be useful to invest in midwifery training, by increasing the capacity of training centers, and setting up simulation centers. Midwives’ working conditions should also be improved, by enhancing their salary conditions and the psychosocial environment in which they work. Finally, it is advisable to continue raising awareness among all stakeholders of the importance of investing in midwifery.

## Discussion

This study conducted a cost-benefit analysis of investing in midwifery in a developing country. The study found that universal coverage of midwifery interventions could save over 120 000 lives by 2030, with an estimated additional investment of US$ 638 288 820. The economic benefit from reduced maternal mortality was US$ 384 860 117, and the benefit from reduced infant mortality was US$ 17 302 052 337. The value added by midwives alone was estimated at US$ 10 152 287 749. The net present value was over US$ 9.5 billion, and the cost-benefit ratio was approximately 16, indicating that the benefits of investing in midwifery outweigh the costs. In addition to the economic benefits demonstrated by our study, investment in midwifery can have wider societal benefits. Several studies have highlighted benefits in terms of relevance and quality of care [18], greater empowerment of women [19], better access to health services [20], reduced health inequalities [21], and better overall health outcomes for women and children [22]. These wide-ranging benefits to the general well-being of society help policymakers and stakeholders understand the overall impact of investing in midwifery beyond the economic perspective.

Our results are consistent with those of the existing literature. Investing in midwifery in LMICs has positive effects on the economy, effectiveness, and efficiency. A cost-effectiveness evaluation conducted in Bangladesh found that the return on investment could be as high as 16 times the amount invested, corresponding to the SoWMy report on midwifery in 73 LMICs. This result is very close to the 15.91 estimated by our study. Nevertheless, the context of the analysis is somewhat divergent, as the benefit estimate in the Bangladesh study was based on the number of caesarean deliveries that could be avoided over 30 years, from 2015 to 2045, through the training and deployment of community midwives. In contrast, the benefit estimate in our study was based on the reduction in maternal, foetal, and neonatal mortality [23]. In both studies, the return on investment was high, indicating that investing in midwives can have a wide effect on the economy as a whole and could be a better alternative investment in primary healthcare. Therefore, investment in midwifery should be considered as an important strategy for improving maternal and child health and achieving the SDGs.

Despite progress in Morocco and other LMICs, challenges remain in improving maternal and child health. Investment in recruiting and training midwives may not be enough, and must be complemented with investment in regulation, effective management of human resources, promotion of a supportive healthcare environment, and staff retention [10]. Financial incentives, such as performance-based payment, could enhance midwives’ productivity and reduce turnover, particularly in areas with low health staff density or for complex medical acts. The state of midwifery practice in Morocco reveals that 15% of midwives left the profession for non-retirement or death reasons in 2014 [24]. Addressing these challenges is essential to ensure continued progress in maternal and child health and to achieve the SDGs.

The study highlights the importance of investing in midwifery in developing countries like Morocco and shows that the benefits outweigh the costs. The study suggests that public policy efforts should be made to increase awareness of the importance of investing in midwifery and to accelerate the process of developing the legal framework for midwifery in Morocco and other LMICs. The investment in midwifery will provide social and health benefits, and meet future demand for health services. The study has some limitations in that it only considers benefits resulting in reduced maternal, stillbirth, and neonatal mortality and does not account for the progression of health workers’ salaries and on-call bonuses. The study results could be particularly relevant for countries economically similar to Morocco.

Several areas of research could contribute to increasing and improving midwifery care in LMICs, better informing maternal and child health policies accordingly. Research could explore the long-term impact of investment in midwifery on health system sustainability. Future research could also investigate the barriers and enablers to midwifery retention and job satisfaction. This will provide further evidence for the ongoing discussion on midwifery investment in LMICs.

## Conclusion

Midwifery is an essential resource for providing SRMNCAH services. However, more work needs to be done to achieve the SDGs, which requires political will and concerted action. Maintaining the advances made in LMICs’ maternal and child health and investing in midwifery is crucial for achieving full coverage of SRMNCAH and ensuring access to high-quality healthcare. Our study showed that increased investment in midwifery is an efficient strategy for achieving these goals and ensuring access to care for LMICs’ population.

## Data Availability

Data that support the study results are available from the Moroccan Ministry of Health and Social Protection, the Lancet series on midwifery care, the World Bank Group’s Morocco economic report, the United Nations Population Fund’s (UNFPA) State of the World’s Midwifery (SoWMy) report, and the World Health Organization’s social discount rate recommendation.

## Abbreviations

CHERG: Child Health Epidemiology Reference Group
FTEs: Full-time equivalents
GDP: Gross domestic product
ICMs: International Confederation of Midwives
LiST: Lives Saved Tool
LMICs: Low- and middle-income countries
SDGs: Sustainable development goals
SoWMy: State of the World’s Midwifery
SRMNCAH: Sexual, reproductive, maternal, new-born, child, and adolescent health
WHO: World Health Organization
